# Effect of Nb and F Co-doping on Li_1.2_Mn_0.54_Ni_0.13_Co_0.13_O_2_ Cathode Material for High-Performance Lithium-Ion Batteries

**DOI:** 10.3389/fchem.2018.00076

**Published:** 2018-04-05

**Authors:** Lei Ming, Bao Zhang, Yang Cao, Jia-Feng Zhang, Chun-Hui Wang, Xiao-Wei Wang, Hui Li

**Affiliations:** ^1^School of Metallurgy and Environment, Central South University, Changsha, China; ^2^Medical Engineering Center, Xiangya Hospital of Central South University, Changsha, China

**Keywords:** Li_1.2_Mn_0.54_Ni_0.13_Co_0.13_O_2_, Nb and F co-doping, cathode material, coulombic efficiency, electrochemical property

## Abstract

The Li_1.2_Mn_0.54−x_Nb_x_Co_0.13_Ni_0.13_O_2−6x_F_6x_ (*x* = 0, 0.01, 0.03, 0.05) is prepared by traditional solid-phase method, and the Nb and F ions are successfully doped into Mn and O sites of layered materials Li_1.2_Mn_0.54_Co_0.13_Ni_0.13_O_2_, respectively. The incorporating Nb ion in Mn site can effectively restrain the migration of transition metal ions during long-term cycling, and keep the stability of the crystal structure. The Li_1.2_Mn_0.54−x_Nb_x_Co_0.13_Ni_0.13_O_2−6x_F_6x_ shows suppressed voltage fade and higher capacity retention of 98.1% after 200 cycles at rate of 1 C. The replacement of O^2−^ by the strongly electronegative F^−^ is beneficial for suppressed the structure change of Li_2_MnO_3_ from the eliminating of oxygen in initial charge process. Therefore, the initial coulombic efficiency of doped Li_1.2_Mn_0.54−x_Nb_x_Co_0.13_Ni_0.13_O_2−6x_F_6x_ gets improved, which is higher than that of pure Li_1.2_Mn_0.54_Co_0.13_Ni_0.13_O_2_. In addition, the Nb and F co-doping can effectively enhance the transfer of lithium-ion and electrons, and thus improving rate performance.

## Introduction

Lithium-ion batteries (LIBs) have been broadly used in the portable electronics, and regarded as the most promising energy storages for hybrid electric vehicles (HEVs) and electric vehicles (EVs) (Tarascon and Armand, 2001; Armand and Tarascon, 2008; Chiang, 2010). It is generally believed that the cathode materials are the primary factors for the improvements of the lithium-ion batteries. However, conventional cathode materials, such as LiCoO_2_, LiMn_2_O_4_, and LiFePO_4_ show low specific capacity and unsatisfied energy density, which will limit further practical application in the energy storage system (Ding et al., 2011; Luo et al., 2012; Zheng et al., 2015a). Among the developed cathode materials, lithium-rich layered material attracts great attentions of scientists, due to their high capacity of above 250 mAh g^−1^, high operating voltage and low cost compared with other cathode materials (Thackeray et al., 2005; Park et al., 2007). It is a pity that lithium-rich layered materials have some intrinsic drawbacks of huge initially irreversible capacity, poor rate capability, the continous capacity and voltage decay during long-term cycling, which block their practical applications (Ellis et al., 2010). The poor rate capability is due to the poor electrical conductivity of Li_2_MnO_3_ component in lithium-rich materials, while the low initial coulombic efficiency is related to the elimination of O^2−^ make the change of structure of Li_2_MnO_3_ in charge process (Johnson et al., 2007; He et al., 2012). Additionally, the capacity and voltage decay is caused by the structure transformation and formation of passivation layer during cycling (Lu and Dahn, 2002; Armstrong et al., 2006).

To solve above problems, many methods are proposed, such as surface coating, ion doping, and particle size reducing. Generally, surface coating could effectively suppress the side reaction between lithium-rich layered material and electrolyte and elimination of the oxygen vacancies, thus improving initial coulombic efficiency and cycling stability (Li et al., 2014, 2015, 2016). The reducing particles size could shorten the pathway to enhance the rate capability (Zheng et al., 2017). However, above approaches cannot effectively suppressed the voltage decay during cycling. Bulk cationic doping with Al, Mg, Cr, Zr, and Ru could effectively suppress the migration of transition metal (TM) during cycling, and mitigate the capacity and voltage decay (Kim et al., 2006; Jiao et al., 2007; Luo and Dahn, 2011; Sathiya et al., 2013; Xu et al., 2014). In addition, O^2−^ site is replaced by anions, such as Cl^−^ and F^−^, which could be beneficial for suppressing the structure change of Li_2_MnO_3_ from the eliminating of oxygen in initial charge process, and thus Li^+^ could return to the material lattice in subsequent charge-discharge process (Kang and Amine, 2005; Park et al., 2005). Therefore, the bulk doping also increase the electronic conductivity, and improve the rate performance of material bulk.

Therefore, we described the incorporation of Nb^5+^ and F^−^ into the Mn site and O site of Li_1.2_Mn_0.54_Ni_0.13_Co_0.13_O_2_ (LMNCO), respectively. The Nb^5+^ and F^−^ co-doping suppress the TM migration during cycling and alleviate Li^+^ loss during the elimination of O^2−^ in the charging process. In this paper, the doped Li-rich layered oxide (Li_1.2_Mn_0.54−x_Nb_x_Co_0.13_Ni_0.13_O_2−6x_F_6x_) is synthesized by high temperature solid phase method, the effects of Nb^5+^ and F^−^ co-doping on the initial coulomb efficiency, rate performance, cycle performance and work voltage are discussed in detail.

## Experimental

### Materials synthesis

**Figure 1:**
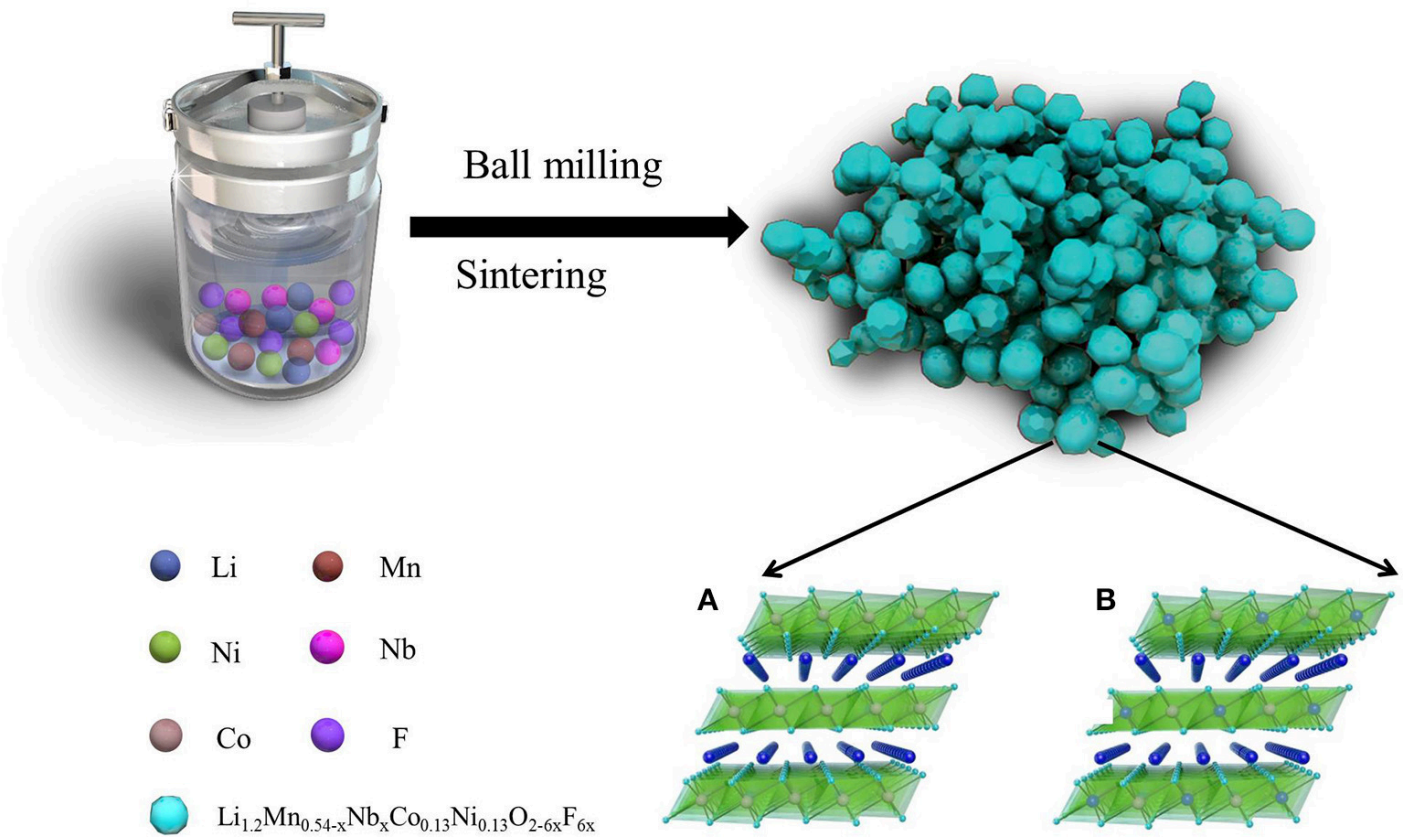
Schematic illustration of the fabrication of Nb and F co-doped Li_1.2_Mn_0.54−x_Nb_x_Co_0.13_Ni_0.13_O_2−6x_F_6x_. Crystal structure of LiMO_2_ **(A)** and Li_2_MnO_3_ **(B)**.

The fabrication process of Li_1.2_Mn_0.54−x_Nb_x_Co_0.13_Ni_0.13_O_2−6x_F_6x_ (*x* = 0, 0.01, 0.03, 0.05) by high temperature solid phase method is schemed in Figure 1. Typically, the stoichiometric Li(CH_3_COO)·2H_2_O, Mn(CH_3_COO)_2_·4H_2_O, Ni(CH_3_COO)_2_·4H_2_O, Co(CH_3_COO)_2_·4H_2_O, Nb_2_O_5_, LiF and citric acid were mixed with 50 wt% of deionized water by ball-milling for 8 h (all chemicals of 99% purity). The mole ratio of Li(CH_3_COO)·2H_2_O, Mn(CH_3_COO)_2_·4H_2_O, Ni(CH_3_COO)_2_·4H_2_O, Co(CH_3_COO)_2_·4H_2_O, Nb_2_O_5_, LiF are 1.2: 0.54-x: 0.13: 0.13: x/2: 6x (*x* = 0, 0.01, 0.03, 0.05), respectively. Then the mixtures were dried at 80°C for 12 h, and ground into fine particles. Finally, the mixture powders were calcined at 550°C for 5 h, follow at 850°C for 15 h in air to get a set of Li-rich layered oxide materials Li_1.2_Mn_0.54−x_Nb_x_Co_0.13_Ni_0.13_O_2−6x_F_6x_. The Li_1.2_Mn_0.54−x_Nb_x_Co_0.13_Ni_0.13_O_2−6x_F_6x_ materials with *x* = 0, 0.01, 0.03, 0.05 are shorted as LMNCO-NF0, LMNCO-NF1, LMNCO-NF3, and LMNCO-NF5, respectively.

### Characterization

All materials were characterized through an X-ray diffraction (XRD: Rigaku, D/max 2500v/pc) with Cu Kα radiation. The scanning electron microscopy (SEM: Philips, FEI Quanta 200 FEG) and transmission electron microscopy (TEM: JEM-2010, JEOL) were applied to observed the microstructure and the structure of all materials. The elemental chemical states of all materials were analyzed by X-ray photoelectron spectroscopy (XPS, PHI 5000VersaProbe).

### Electrochemical evaluation

Electrochemical performance of all Li-rich layered oxide Li_1.2_Mn_0.54−x_Nb_x_Co_0.13_Ni_0.13_O_2−6x_F_6x_ were tested using CR2032 coin cell. The electrode preparation process was consisted of three steps as follow. Firstly, 80% active material (LMNCO), 10% acetylene black, and 10% polyvinylidene fluoride (PVDF) binder were mixed with NMP solvent. Secondly, as prepared viscous cathode slurry was cast on aluminum foil. Thirdly, the foil was dried at 90°C under vacuum for 12 h. Then it was punched into 12 mm diameter disks with the loading of active cathode mass in the range of 3–4 mg cm^−2^. The coin cells were assembled in an argon-filled dry box. The lithium metal and the Celgard 2500 were used as anode material and the separator, respectively. 1 M LiPF_6_ in ethylene carbonate/diethyl carbonate (*V*/*V* = 1:1) was used as electrolyte. The galvanostatic charge-discharge measurements were carried out on LAND CT2001A battery testing system (Wuhan, China). Cyclic voltammetry (CV) measurements were performed by IM6 electrochemical testing station at scan rates of 0.1 mV s^−1^ between 2.0 and 4.8 V. Electrochemical impedance spectroscopy (EIS) was conducted by IM6 electrochemical testing station between 100 kHz and 0.01 Hz by applying perturbation AC voltage signal of 5 mV.

## Results and discussion

**Figure 2:**
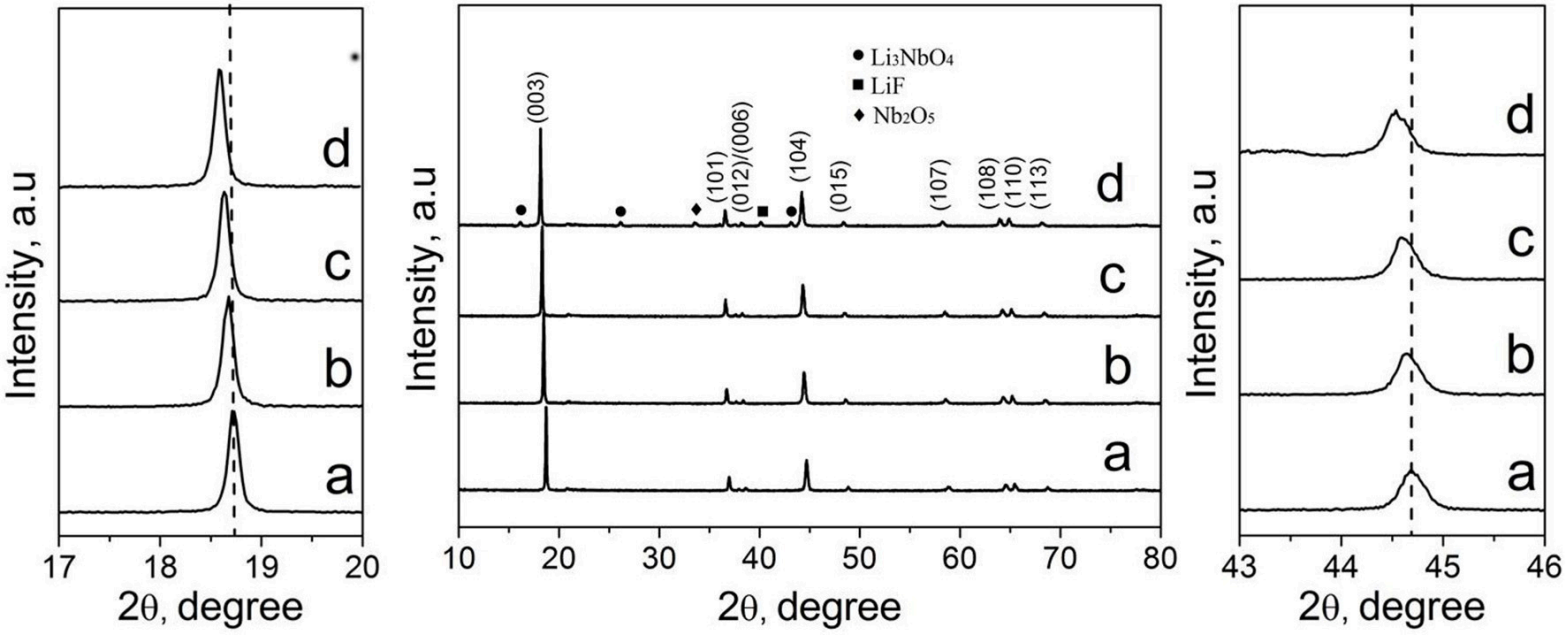
XRD patterns of (a) LMNCO-NF0, (b) LMNCO-NF1, (c) LMNCO-NF3, and (d) LMNCO-NF5, and the magnified view of peak (003) and (104).

Figure 2 shows the XRD patterns of Li_1.2_Mn_0.54−x_Nb_x_Co_0.13_Ni_0.13_O_2−6x_F_6x_ materials (*x* = 0, 0.01, 0.03, and 0.05). As seen Figure S1A, the XRD pattern of LMNCO-NF0 material belongs to the layered α-NaFeO_2_ structure with space group R3m (Figure S1A). There is a weak diffraction peak around 20–25° in the XRD pattern of the LMNCO-NF0, corresponding to the short-range cation ordering of Li^+^ and Mn^4+^ in the transition metal layers, as illustrated for Li_2_MnO_3_ structure in Figure S1B (Jarvis et al., 2011). The adjacent peaks of (006)/(012) and (108)/(110) show obvious separation, indicating the perfect layer structure of LMNCO-NF0 (Gong et al., 2004). Meanwhile, the intensity ratio of I(003)/I(104) is the indication of mixing degree for transition-metal ions in the lithium layer (Zheng et al., 2015b). For LMNCO-NF0, the I(003)/I(104) value reach 1.6, suggesting low mixing degree of transition-metal ions in the lithium layer. After doping, the XRD patterns of all samples are similar to that of LMNCO-NF0, and adjacent peaks of (006)/(012), (108)/(110) and I(003)/I(104) value remain significantly unchange. The XRD patterns of LMNCO-NF1 and LMNCO-NF3 show highly pure phase, implying that Nb and F ions are successfully doped into the crystal lattice. But when the doping amount increases, the impurity phases of Li_3_NbO_4_, Nb_2_O_5_, and LiF are observed in the XRD pattern of LMNCO-NF5, due to the solid solubility of Nb and F elements in the Li_1.2_Mn_0.54_Co_0.13_Ni_0.13_O_2_ material is beyond the limitation. In addition, for all doped materials, the diffraction peaks of doped samples slightly shift to lower 2θ compared to that of the LMNCO-NF0, indicating that Nb and F doping can enlarge the interlayer spacing. Furthermore, the lattice parameters of all samples are calculated by Rietveld refinement, and the results are listed in Table 1. It is clearly seen that the values of lattice parameter a and c get higher along with increasing amount of Nb and F ion doping. While the increasing ratio of c/a for doped sample represents low mixing degree for transition-metal ions in the lithium layer. This phenomenon also suggests that cell volume is enlarged after doping with Nb^5+^ and F^−^, which is beneficial for the diffusion of the Li^+^ ions (Jafta et al., 2012). In addition, the average crystallite size of the LMNCO-NF0, LMNCO-NF1, LMNCO-NF3, and LMNCO-NF5 are calculated by using Scherrer equation [βcos(θ) = kλ/D], where β is full-width at half-maximum (FWHM) of the XRD peak and k is a constant (0.9) as given in Table S1. This suggests that the addition of Nb_2_O_5_ and LiF could suppressed the growth of crystallite size.

**Table 1:** The crystallographic parameters of LMNCO-NF0, LMNCO-NF1, LMNCO-NF3, and LMNCO-NF5, respectively.

**Samples**	**a (Å)**	**c (Å)**	**c/a**
LMNCO-NF0	2.8515	14.2263	4.9863
LMNCO-NF1	2.8536	14.2352	4.9869
LMNCO-NF3	2.8546	14.2396	4.9875
LMNCO-NF5	2.8555	14.2457	4.9889

In order to analyze the effect of Nb and F co-doping on chemical composition of LMNCO, the stoichiometric amounts of metal element in all samples have been determined by ICP analysis, and the result are listed in Table S2. As seen in Table S2, the molar ratio of Li: Ni: Co: Mn in pristine LMNCO is 1.213: 0.133: 0.132: 0.543, which is close to the theoretical ratio of 1.2: 0.133: 0.133: 0.54. The molar ratio of Li: Ni: Co: Mn of all doped samples (LMNCO-NF1, LMNCO-NF3, and LMNCO-NF5) does not vary compare to pristine LMNCO, suggesting that the Nb and F co-doping not affect the chemical composition of the samples.

The SEM images of Li_1.2_Mn_0.54−x_Nb_x_Co_0.13_Ni_0.13_O_2−6x_F_6x_ materials (*x* = 0, 0.01, 0.03, and 0.05) are shown in Figure S2. The particle sizes of all doped samples (LMNCO-NF1, LMNCO-NF3, and LMNCO-NF5) are slightly smaller than that of LMNCO-NF0. Particularly, the particles sizes of doped samples decrease with the increasing amount of Nb^5+^ and F^−^ elements, suggesting Nb_2_O_5_ and LiF can exhibit space steric effect, thus effectively suppressing the growth of particles. The tap density of pure LMNCO and all doped samples (LMNCO-NF1, LMNCO-NF3, and LMNCO-NF5) are listed in Table S3. The tap density the pure LMNCO and all doped samples (LMNCO-NF1, LMNCO-NF3, and LMNCO-NF5) are 1.51, 1.50, 1.52, and 1.49, respectively. The tap density of all doped samples (LMNCO-NF1, LMNCO-NF3, and LMNCO-NF5) not vary compared to pure LMNCO. In addition, EDS elements mapping test has been performed on the LMNCO-NF3. As seen Figure S2E, the element mappings clearly demonstrate that Ni, Co, Mn, Nb, and F elements are all homogeneously distributed in LMNCO-NF3 structure, which confirm that Nb and F are doped into the bulk material.

**Figure 3:**
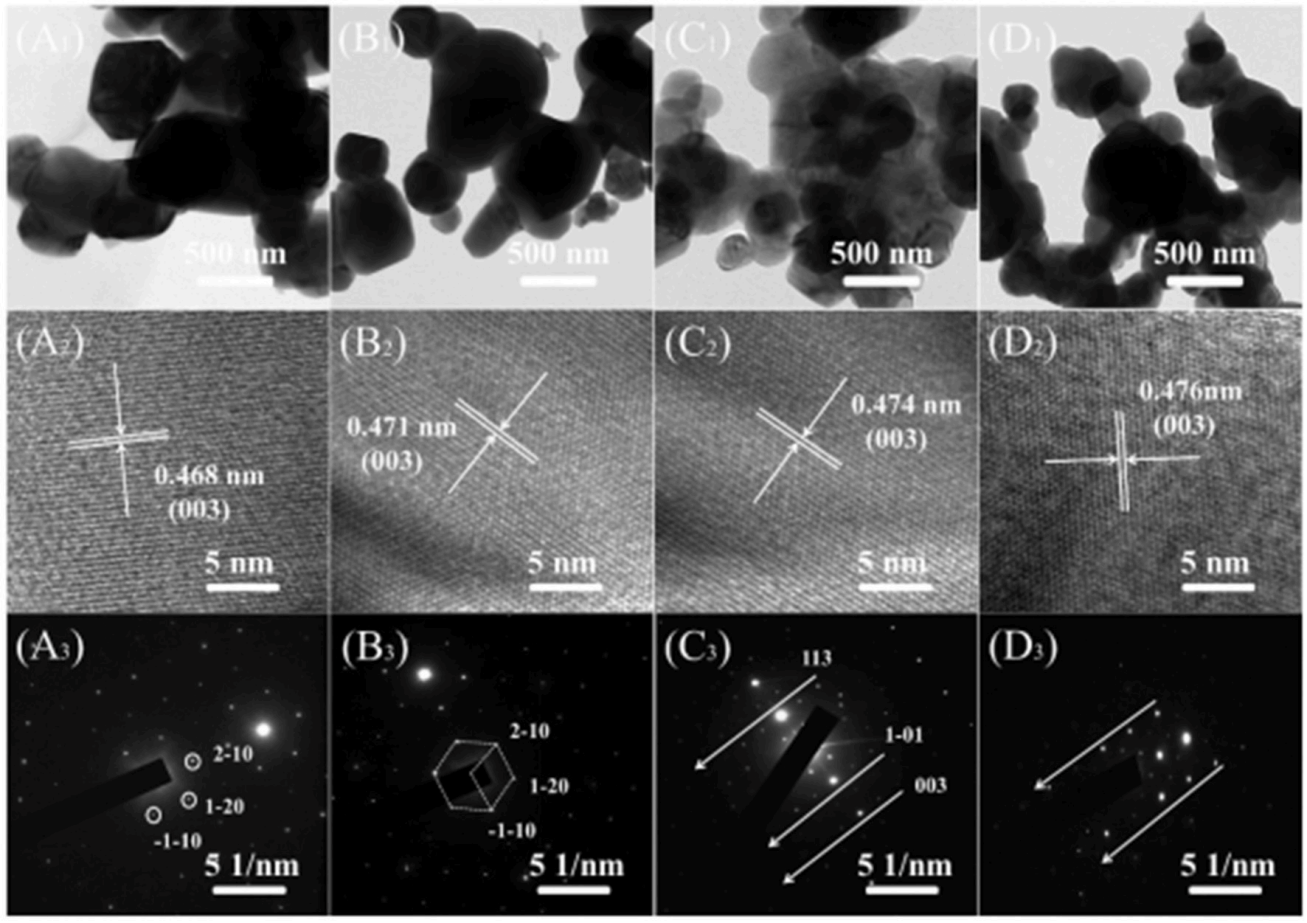
TEM, HRTEM images and SEAD patterns of **(A**_1_**-A**_3_**)** LMNCO-NF0, **(B**_1_**-B**_3_**)** LMNCO-NF1, **(C**_1_**-C**_3_**)** LMNCO-NF3, and **(D**_1_**-D**_3_**)** LMNCO-NF5, respectively.

Figure 3 show TEM, HETEM and selected area electron diffraction (SAED) patterns of the LMNCO-NF0, LMNCO-NF1, LMNCO-NF3, and LMNCO-NF5. The interplanar spacing of the lattice fringes (003) gradually expand along with the increase content of Nb and F co-doping, which is consistent with the XRD calculated results. This phenomenon is ascribed to that the Nb^5+^ ions doped into the Mn^4+^ sites and the F^−^ ions occupy the pack oxygen sites. The (SAED) patterns (Figures 3A_3_-D_3_ reveal that all the samples belong to the hexagonal symmetry of the local structure.

**Figure 4:**
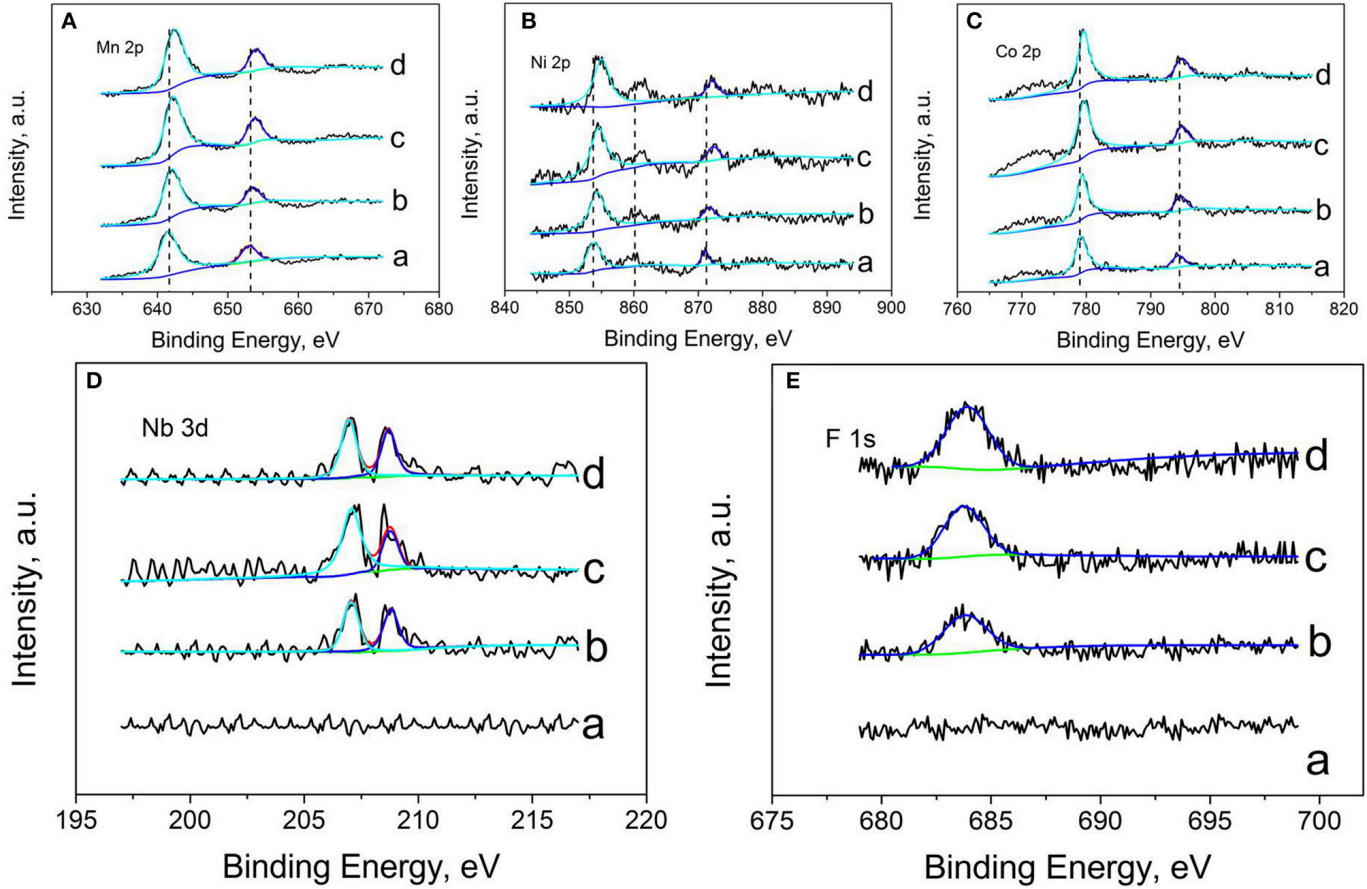
XPS patterns of **(A)** Mn 2p, **(B)** Ni 2p, **(C)** Co 2p, **(D)** Nb 3d, and **(E)** F 1s of (a) LMNCO-NF0, (b) LMNCO-NF1, (c) LMNCO-NF3, and (d) LMNCO-NF5.

In order to investigate the effect of Nb and F ion co-doping on the oxidation states of some elements (Ni, Mn, Co, Nb, F, O) for all samples, Figure 4 shows the XPS spectra of Li_1.2_Mn_0.54−x_Nb_x_Co_0.13_Ni_0.13_O_2−6x_F_6x_ materials (*x* = 0, 0.01, 0.03, and 0.05), respectively. For the doped samples, the Ni, Mn, Co binding energy peak all shift to higher binding energy compare to that of LMNCO-NF0, resulting from the density of electron clouds reduce around LMNCO. The Nb^5+^ ion doping into Mn^4+^ site will reduce the electron clouds. The electronegative of F^−^ is stronger than that of O^2−^, and the electron clouds of all transition metal elements tend to bond with F^−^, suggesting that the F^−^ is successfully doping into the site of pack oxygen. The F 1s and Nb 3d binding energy peaks are not detected in XPS spectrum of LMNCO-NF0. However, after doped Nb and F elements, it is obviously observed the binding energy peak of F 1s and Nb 3d for the LMNCO-NF1, LMNCO-NF3, and LMNCO-NF5. The peak intensities of F1s and Nb 3d increase along with the increment amount of the Nb and F doping. The above analysis suggests that Nb and F successfully doped into the Mn site and O site of Li_1.2_Mn_0.54_Co_0.13_Ni_0.13_O_2_.

**Figure 5:**
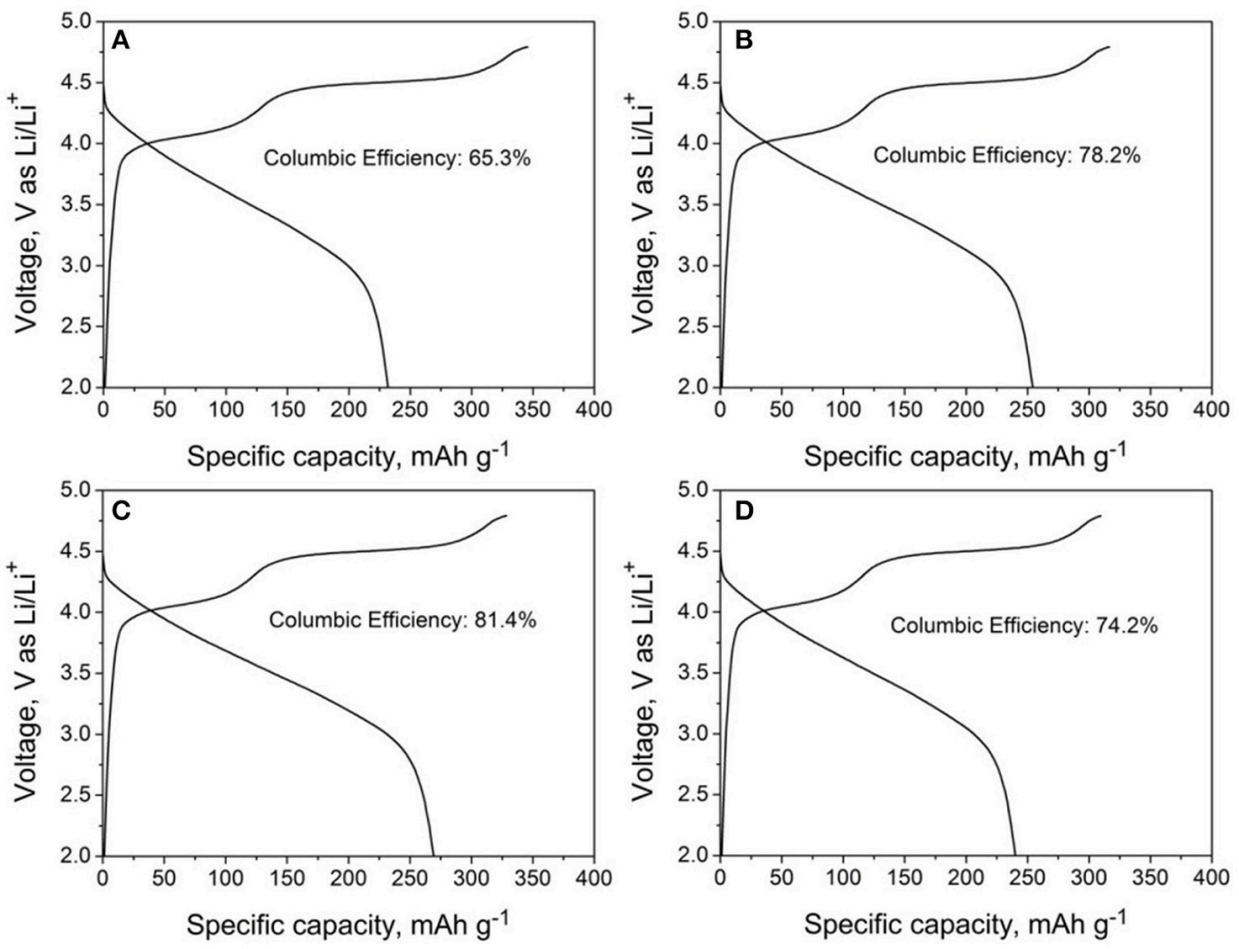
The initial cycle charge-discharge curves of **(A)** LMNCO-NF0, **(B)** LMNCO-NF1, **(C)** LMNCO-NF3, and **(D)** LMNCO-NF5 between 2.0 V and 4.8 V at the current rate of 0.1 C.

The initial charge/discharge curves of LMNCO-NF0, LMNCO-NF1, LMNCO-NF3, and LMNCO-NF5 between 2.0 V and 4.8 V at 0.1 C. are showed in the Figure 5. All samples exhibit two plateaus. One plateau below 4.5 V is related to lithium extraction form the layered LiMO_2_, and the other plateau above 4.5 V corresponds to the lithium-ion extraction from the Li_2_MnO_3_ component and accompanied by the extraction of oxygen (Johnson et al., 2004). The charge/discharge capacities of LMNCO-NF0, LMNCO-NF1, LMNCO-NF3, and LMNCO-NF5 are 354.8/231.8, 325.1/254.1, 337.3/269.3, and 318.9/239.9, respectively. Therefore, for LMNCO-NF1, LMNCO-NF3, and LMNCO-NF5, the initial coulombic efficiency reaches 78.2, 79.84, and 75.2%, respectively, which are higher than that of LMNCO-NF0(65.3). Furthermore, as can be seen in Table S6, the coulombic efficiency of LMNCO-NF3 is better than these previously reported articles (Wang et al., 2013; Chao et al., 2014; Jin et al., 2014; He et al., 2015; Yin et al., 2015). It is confirmed that the covalency of the metal-oxygen bond and electronegativity of the dopant ions have significant influence on the degree of oxygen loss from the lattice, easily mitigating the structure change of the Li_2_MnO_3_ during charging process (Wang and Manthiram, 2013). Therefore, the initial coulombic efficiency is improved by Nb and F effective co-doping.

**Figure 6:**
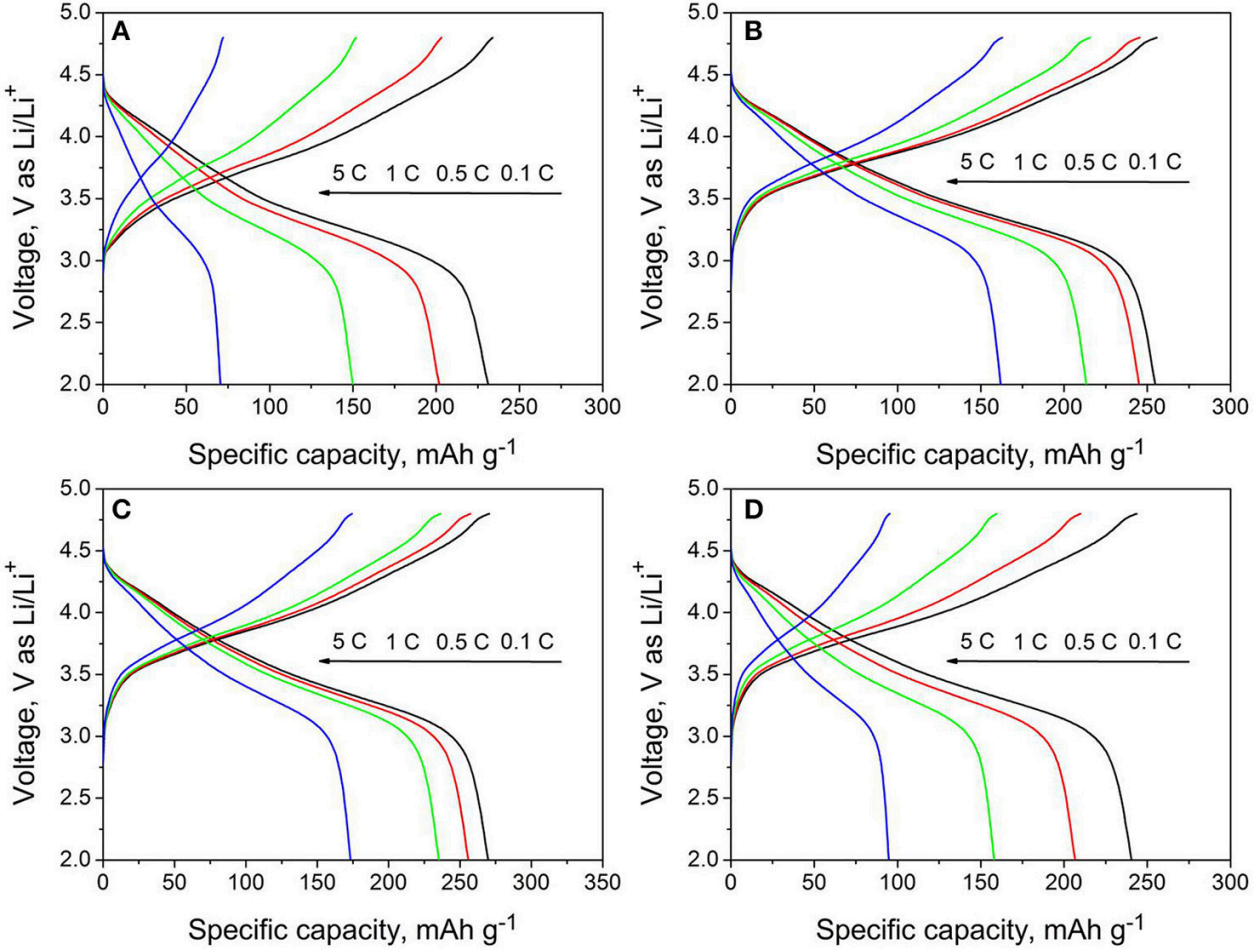
The rate performances of **(A)** LMNCO-NF0, **(B)** LMNCO-NF1, **(C)** LMNCO-NF3, and **(D)** LMNCO-NF5 between 2.0 V and 4.8 V from 0.1C to 5C.

Figure 6 shows the charge/discharge curves of all samples at different rates of 0.1, 0.5, 1, and 5C. It is seen that the rate performance is enhanced by moderate amount of Nb and F co-doping. The discharge capacities of LMNCO-NF1 are 254.8, 245.2, 213.5, 162 mAh g^−1^ at 0.1 C, 0.5 C, 1 C, and 5 C, while the discharge capacities of LMNCO-NF3 are 269.8, 257.3, 235.3, and 173.3 mAh g^−1^ respectively, which are higher than that of LMNCO-NF0 (231.2, 201.9, 149.9, and 70.4 at 0.1C, 0.5C, 1C, and 5C, respectively). The improved rate performance is related to the inequitable valent doping of Nb and F element in Mn and O sites, respectively, which can increase the oxygen vacancies in material surface, enhancing the electronic conductivity of host material eventually. However, the discharge capacities of LMNCO-NF5 are 240.6, 206.7, 158, and 94.8 mAh g^−1^ at 0.1C, 0.5C, 1C, and 5C, respectively. The LMNCO-NF5 show relatively poor rate performance and lower initial coulombic efficiency, owing to that the excessive Nb and F co-doping can form a thick Li_3_NbO_4_/Nb_2_O_5_/LiF layer, and lengthen the lithium ion diffusion path.

**Figure 7:**
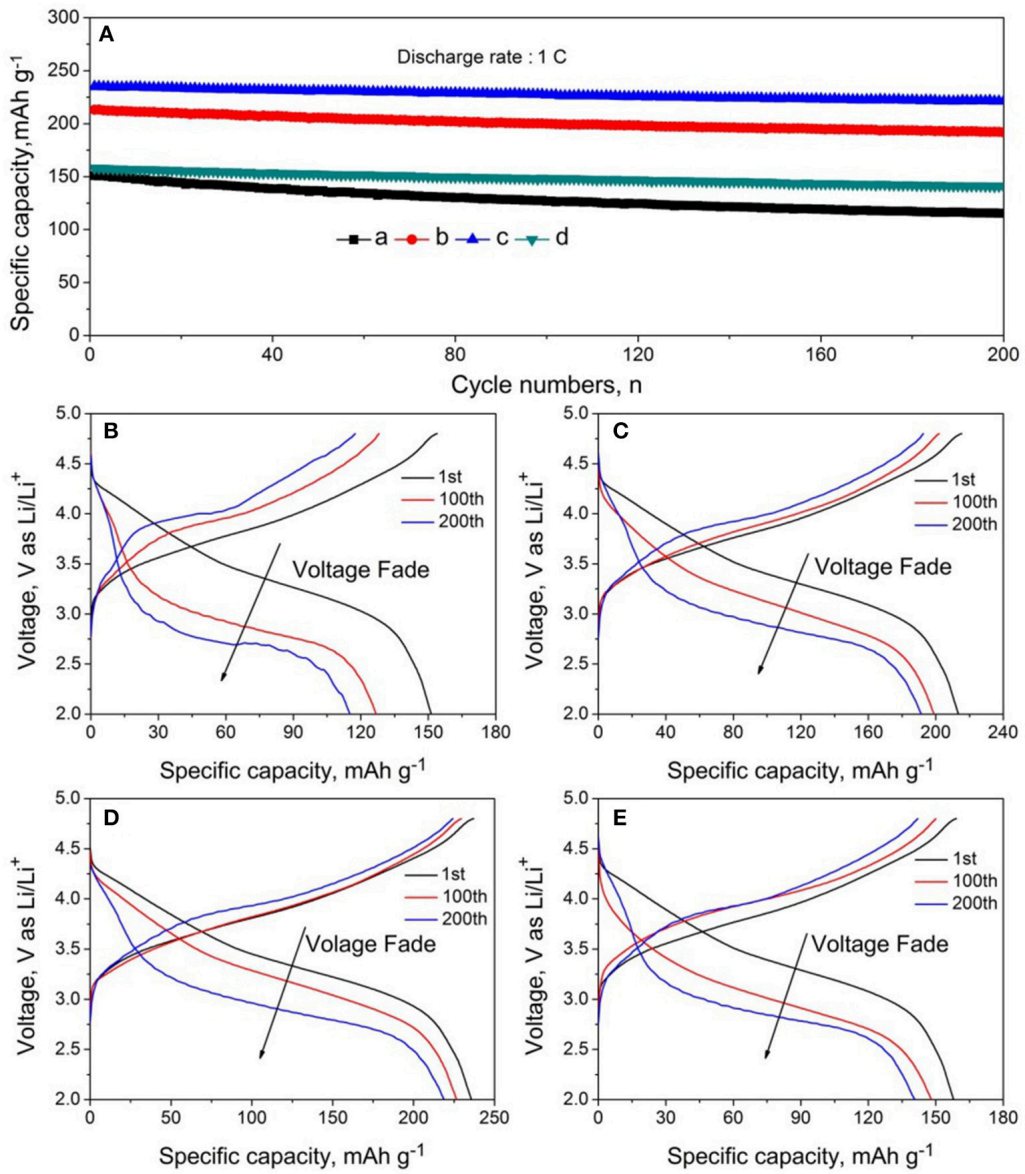
**(A)** Cycling performance and **(B–E)** charge-discharge curves of batteries based on LMNCO-NF0, LMNCO-NF1, LMNCO-NF3, and LMNCO-NF5 cathode materials at 1 C between 2.0 and 4.8 V (vs. Li/Li^+^) at room temperature.

Figure 7 shows the cycling performance and selected charge-discharge cures of LMNCO-NF0, LMNCO-NF1, LMNCO-NF3, and LMNCO-NF5, respectively. The discharge capacity of LMNCO-NF0 is only 115.6 mAh g^−1^ with capacity retention of only 76.5% after 200 cycles at 1 C. In comparison, the discharge capacity of LMNCO-NF1, LMNCO-NF3, and LMNCO-NF5 are 191.8, 221.5, and 140.9 mAh g^−1^ with capacity retention of 88.3, 94.2, and 89.3%, respectively, which are higher than that of LMNCO-NF0. The fading capacity of LMNCO-NF0 is attributed to the side reaction between organic electrolyte and electrode to form inactive surface layers, and the unfavorable structure change during cycling process. As surface corrosion of the sample can trigger the dissolved Mn, resulting in the capacity fade, Nb and F co-doping can effectively suppress Mn dissolution (Table S4), stabilize the surface structure. The improved cycling performance is ascribed to Nb and F co-doping. Specifically, the binding between O and Nb (Table S5), which is stronger than the Mn-O bond, and the stronger binding of Nb-O can effectively suppress the loss of oxygen from the lattice (Deng and Manthiram, 2011). Meanwhile the strongly electronegative of F^−^ ions can keep the structure stability of Li_2_MnO_3_. Partial substitution of O^2−^ anions by F^−^ is also proposed as a way to stabilize the layered structures with the formation of strong M-F bonds (Li et al., 2015).

**Figure 8:**
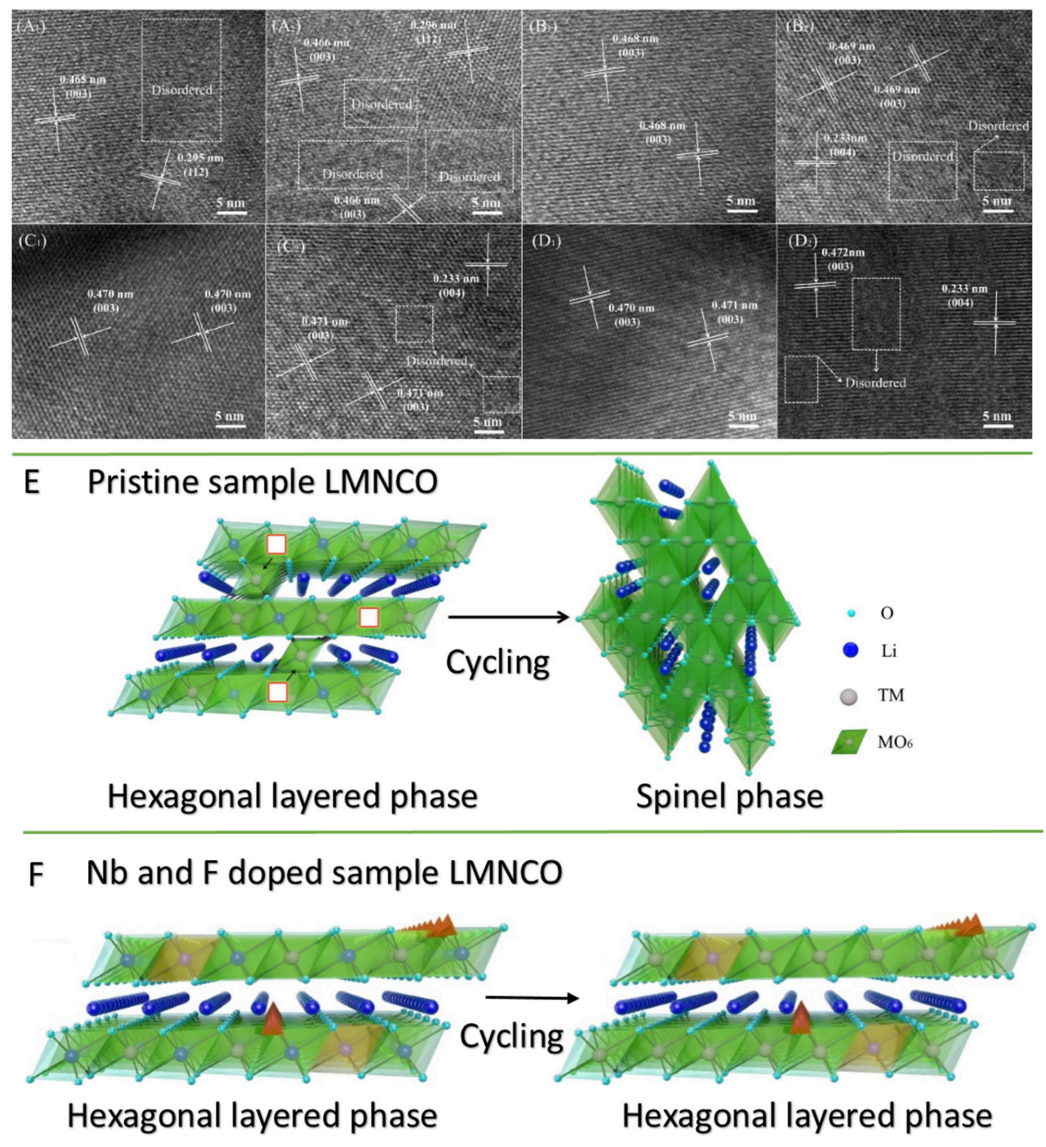
HRTEM images of **(A1,A2)** LMNCO-NF0, **(B1,B2)** LMNCO-NF1, **(C1,C2)** LMNCO-NF3, and **(D1,D2)** LMNCO-NF5 after 100 and 200 cycles. Schematic drawing illustrates the phase transformation for **(E)** pristine and **(F)** Nb and F doped LMNCO layered oxides in the transition layer.

The inhibitory effect of structural transformation of Nb and F co-doped could be seen from the charge-discharge curves. As seem Figure 7B, the corresponding discharge profile of LMNCO-NF0 at 1C exhibited obvious voltage fade after cycling process. The average voltage only is 2.83 V with the average voltage of 77.5% at first cycle after 200 cycles. During the long-term cycling process, the migration of transition metal ions will result in the transformation of layered structure to spinel structure, accompanied by the continous voltage fade as the consequence (Figure 8E). While, after Nb and F co-doping, the voltage decay are effectively suppressed. The average voltages of LMNCO-NF1, LMNCO-NF3, and LMNCO-NF5 can reach 3.12 V, 3.23 V, and 3.01 V after 200 cycles, respectively, The Nb^5+^ and F^−^ doped into the bulk material which could suppress the migration of TM ions to Li layer, and thus suppress the layered to spinel phase transformation (Figure 8F).

In order to further confirm the effect of Nb and F co-doping on the electrochemical performance, the cyclic voltammetry (CV) measurements from 2.0 to 4.8 V at a scan rate of 0.1 mVs^−1^ were carried out, as shown in Figure S3. For all samples, there are two anodic peaks at 4.08 and 4.62 V during the initial cycle. The anodic peak at 4.08 V belongs to lithium deintercalation from LMO structure accompanied by the oxidation of Ni^2+^/Ni^4+^ and Co^3+^/Co^4+^, and the other peak at 4.62 V corresponds to lithium deintercalation from Li_2_MnO_3_ structure, which is associated with the Mn^4+^ activation process (Yu and Zhou, 2012). However, the anodic peak at high potential of 4.62 V is ascribed to the irreversible reaction about removal of Li_2_O from the Li_2_MnO_3_ component, which will disappear in the following cycles (Ohzuku et al., 2011). During the reduction process, the cathodic peak at 3.75 V corresponds to the redcution of Ni^2+^/Ni^4+^ and Co^3+^/Co^4+^, and the activation of the Li_2_MnO_3_ component. there is the peak at about 3.20 V in following cathodic reaction (Jin et al., 2014). In the subsequent cycles, the peak at 4.6 and 3.9 V of all materials disappears, and the peak at 3.7 V emerges. In addition, for LMNCO-NF0, the reduction peak below 3.0 V is observed after 100 cycles, and the intensity gradually increase with the increase of the cycle, suggesting the formation of the spinel phase during the cycling. However, the reduction peak below 3.0 V for LMNCO-NF1, LMNCO-NF3, and LMNCO-NF5 are hardly observed after 100 cycles, and the reduction peak below 3.0 V appeared when the number of cycles up to 200 cycles. This result indication that the phase transformation from layered into spinel structure is effectively suppressed after Nb and F co-doping.

The results of HRTEM investigations of LMNCO-NF0, LMNCO-NF1, LMNCO-NF3, and LMNCO-NF5 after 100 and 200 cycles are displayed in Figure 8. As seen Figure 8A, the interplanar spacing of LMNCO-NF0 after 100 and 200 cycles show (003) planes of layer phase, and the (112) planes of the spinel phase. Moreover, LMNCO-NF0 was found to the formation of local amorphous and spinel phase domains after 100 cycles, and the local amorphous areas increase after 200 cycles. In contrast local amorphous domains after 100 cycles do not appear. While local amorphous domains and spinel phase of all doped materials after 200 cycles were observed. Meanwhile the local amorphous areas of all doped materials after 200 cycles are less than that of LMNCO-NF0. Furthermore, the (220) and (440) planes of the spinel phase not be observed after 100 cycles, but after 200 cycles emerges. The Nb and F co-doping can effectively mitigate the migration of TM ions, and suppressed the voltage fade during high voltage cycling.

**Figure 9:**
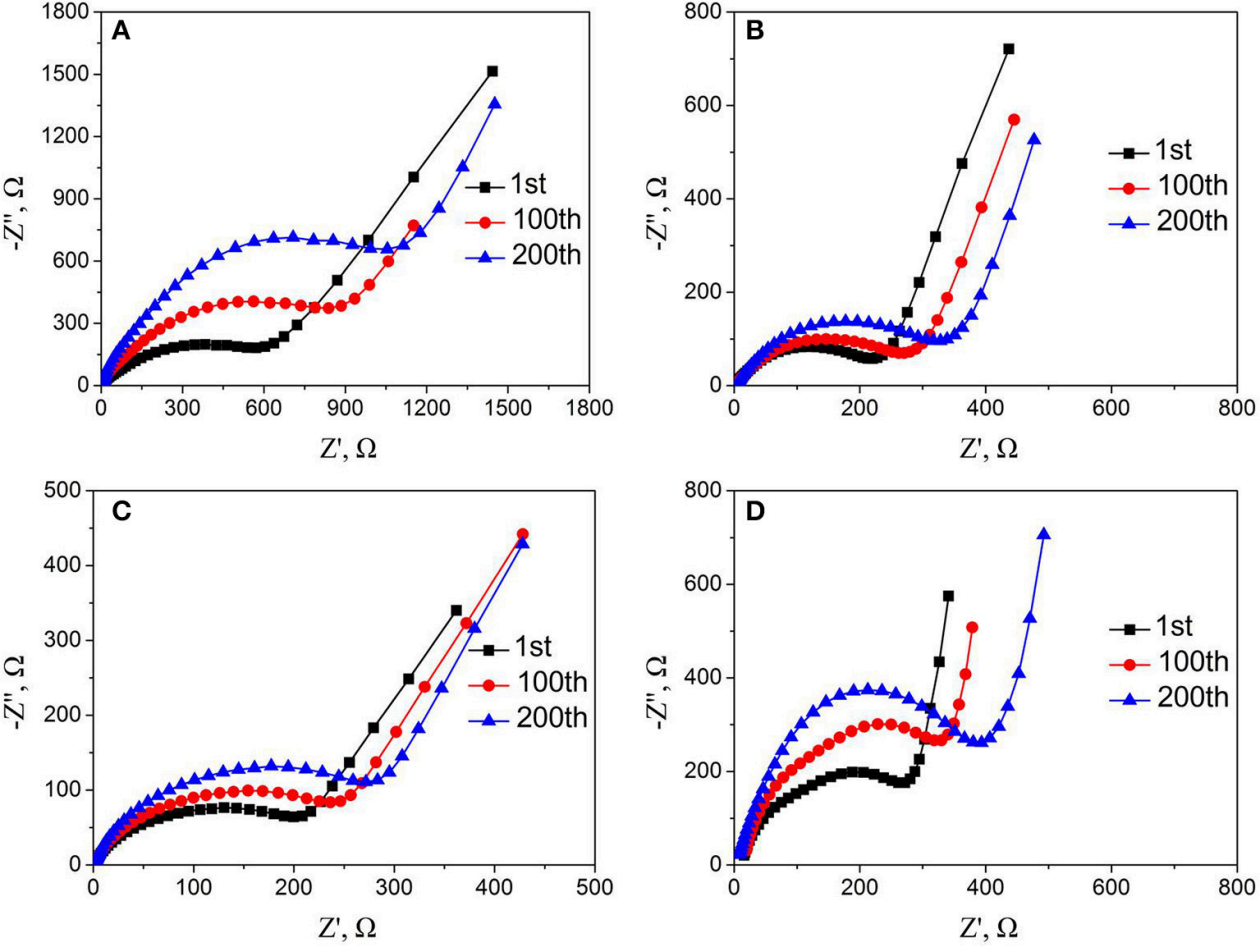
Nyquist plots of **(A)** LMNCO-NF0, **(B)** LMNCO-NF1, **(C)** LMNCO-NF3, **(D)** LMNCO-NF5 electrodes after different cycles at discharge rate of 1C.

To gain insight into the effect of Nb and F co-doping on kinetic behavior of lithium ion diffusion, the EIS was carried out. Figure S4 and Figure 9 show Nyquist plots of all samples before and after cycling, respectively. All the Nyquist plots are consist of the depressed semicircles and a slope. The high frequency intercept at the real axis belongs to the ohmic resistance (R_Ω_) of interaction between electrolyte and electrode, and the semicircle in the middle frequency region corresponds to charge-transfer resistance (R_ct_). The slope in the low frequency region is related to the diffusion of lithium ion in the bulk material (Koga et al., 2013; La Mantia et al., 2013; Wang et al., 2015, 2017). The values of R_ct_ and R_Ω_ of all materials before cycling are simulated by the equivalent circuit and the result were listed in Table 2. In addition, the lithium-ion diffusion coefficients of all samples are calculated by the following equations (Fey et al., 2001; Levi and Aurbach, 2004; Lin et al., 2013) and also listed in Table 2.

(1) $${{\text{D}}_{\text{Li}}}^{+}=\frac{{\text{R}}^{2}{\text{T}}^{2}}{{2\text{n}}^{4}{\text{F}}^{4}{\text{C}}_{\text{Li}}^{2}\sigma^{2}}$$

(2) $$Z^{\prime }=R_{\Omega }+R_{\text{ct}}\sigma \omega^{-1/2}$$

**Table 2:** Impedance parameters derived using equivalent circuit model (Figure S4B) for LMNCO-NF0, LMNCO-NF1, LMNCO-NF3, and LMNCO-NF5 electrodes before cycling (fully discharged).

**Samples**	**LMNCO-NF0**	**LMNCO-NF1**	**LMNCO-NF3**	**LMNCO-NF5**
R_Ω_/Ω	8.3	4.8	4.3	5.2
R_ct_/Ω	573.2	211.3	189.5	243.2
${\text{D}}_{\text{Li}}^{+}$/cm^2^ s^−1^	0.85 × 10^−11^	2.34 × 10^−11^	2.61 × 10^−11^	2.04 × 10^−11^

Here, T is 298 K, R is gas constant (8.314 J K^−1^ mol^−1^), A is the surface area of the electrode, F is the Faraday constant (96,485 C mol^−1^), n is the number of electrons involved in reaction, C is the concentration of lithium ion. Where ω is the angular frequency in the low frequency region and σ is the Warburg coefficient. The graph of Z′ against ω^−1/2^ in the low frequency region is a straight line with the slope of σ.

As seen Table 2, the R_ct_ of LMNCO-NF1, LMNCO-NF3, and LMNCO-NF5 are much lower than that of LMNCO-NF0, suggesting that the Nb and F co-doping will increase electronic conductivity of bulk material and improve the kinetics of lithium-ion diffusion, due to Li slab space is enlarged. Therefore, all doped materials have higher capacity and excellent rate performance.

Meanwhile, further explain the effect of the Nb and F doped on the cycling performance were performed by EIS after the 1st, 100th, and 200th cycles. For all samples, the value of R_ct_ is simulated by the equivalent circuit and the lithium-ion diffusion coefficients are calculated by the equation (1 and 2), while the results are listed in the Table S7. The charge transfer resistance (R_ct_) of LMNCO-NF0 increases continuously, while lithium ion diffusion coefficient (D_Li+_) decreases rapidly and remains only half of the pristine value after 200 cycles, resulting from the severe structure change during long-term cycling. While the charge transfer resistance (R_ct_) and lithium ion diffusion coefficient (D_Li+_) of all doped materials exhibit a little variation and keep acceptable values after 200 cycles, indicating the excellent reaction kinetic. These suggest that Nb and F co-doping could keep the capacity stability which could be ascribed to that doping of Nb and F ions into the bulk layered materials could suppress the change structure from a layered into a spinel structure during cycling.

## Conclusion

Nb and F co-doped Li_1.2_Mn_0.54_Ni_0.13_Co_0.13_O_2_ have been fabricated by using traditional solid phase method. The Nb^5+^ and F^−^ ions are successfully doping into the Mn^4+^ site and O^2−^ sites, respectively, which is beneficial for suppress the loss of the oxygen and the mixed migration of transition metal ions. Therefore, Nb and F co-doping can enhance initial coulombic efficiency of 81.4% and rate performance with discharge capacity with 269.8, 257.3, 235.3, and 173.3mAh g^−1^ at the discharge rates of 0.1, 0.5, 1 C, 5 C and cycling performance with discharge capacity of 221.5 mAh g^−1^ after 200 cycles at 1 C, as well as suppress voltage fade of Li_1.2_Mn_0.54_Ni_0.13_Co_0.13_O_2_ during cycling. It is convinced that the Li_1.2_Mn_0.54_Ni_0.13_Co_0.13_O_2_ after Nb and F co-doping can satisfy the requirements of the electric vehicle and the renewable energy storage, and become advanced lithium ion cathode materials for application of Li-ion battery.

## Author contributions

LM: Designer of the scheme and main performer of the experiment. BZ: Main advisor. YC: Provide assistance in characterization. J-FZ: Main advisor and participant. C-HW: Participant of the experiment. X-WW: Provide assistance in experiment. All authors listed, have made substantial, direct and intellectual contribution to the work, and approved it for publication.

### Conflict of interest statement

The authors declare that the research was conducted in the absence of any commercial or financial relationships that could be construed as a potential conflict of interest.
