# Supplementary material for: Effect of Nb and F Co-doping on Li1.2Mn0.54Ni0.13Co0.13O2 Cathode Material for High-Performance Lithium-Ion Batteries
Source: Front Chem. 2018 Apr 5;6:76. doi: 10.3389/fchem.2018.00076 (PMC5896303; doi:10.3389/fchem.2018.00076)
Supplement: Supplementary file 1 [file Table2.DOC]

Support information

**Effect of Nb and F co-doping on Li_1.2_Mn_0.54_Ni_0.13_Co_0.13_O_2_ cathode material for high-performance lithium-ion batteries**

^a^ *School of Metallurgy and Environment ,Central South University, Changsha 410083, P.R. China;*

* E-mail: yiyzjf@csu.edu.cn

**Table S1**. Average crystal size of all samples (LMNCO-NF0,LMNCO-NF1, LMNCO-NF3 and LMNCO-NF5 prepared with different organic acids at 750, 850 and 950^o^C for 15h, respectively.

| Samples | LMNCO-NF0 | LMNCO-NF1 | LMNCO-NF3 | LMNCO-NF5 |
| --- | --- | --- | --- | --- |
| Average crystal size (nm) | 28 | 26 | 25 | 21 |

**Table S2.** The ICP analysis of LMNCO-NF1 and all doped samples (LMNCO-NF1, LMNCO-NF3 and LMNCO-NF5).

| Samples | Molar ratio of Li: Ni: Co: Mn | | | |
| --- | --- | --- | --- | --- |
|  | Li | Ni | Co | Mn |
| Pristine LMNCO | 1.213 | 0.133 | 0.132 | 0.543 |
| LMNCO-NF1 | 1.211 | 0.132 | 0.131 | 0.541 |
| LMNCO-NF3 | 1.214 | 0.133 | 0.129 | 0.542 |
| LMNCO-NF5 | 1.215 | 0.135 | 0.131 | 0.543 |


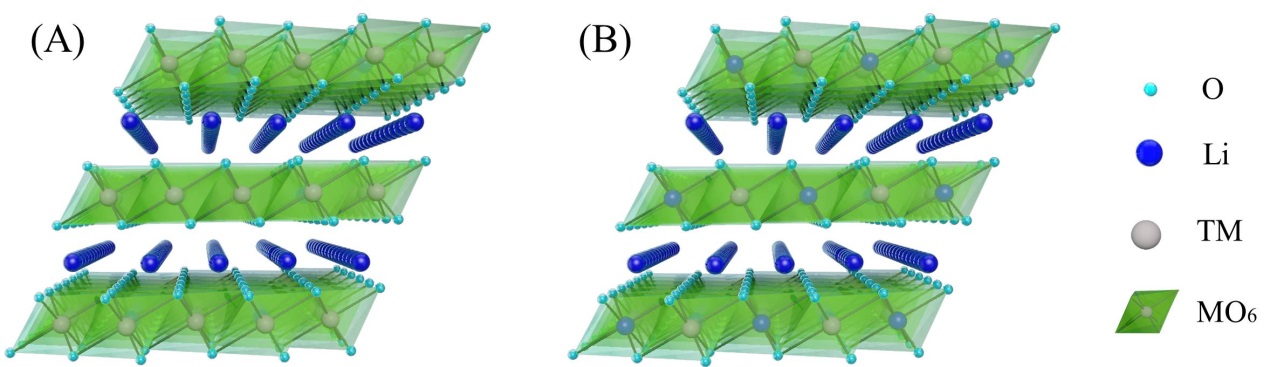


**Fig. S1**. Crystal structure of LiMO_2_ (A) and Li_2_MnO_3_ (B).


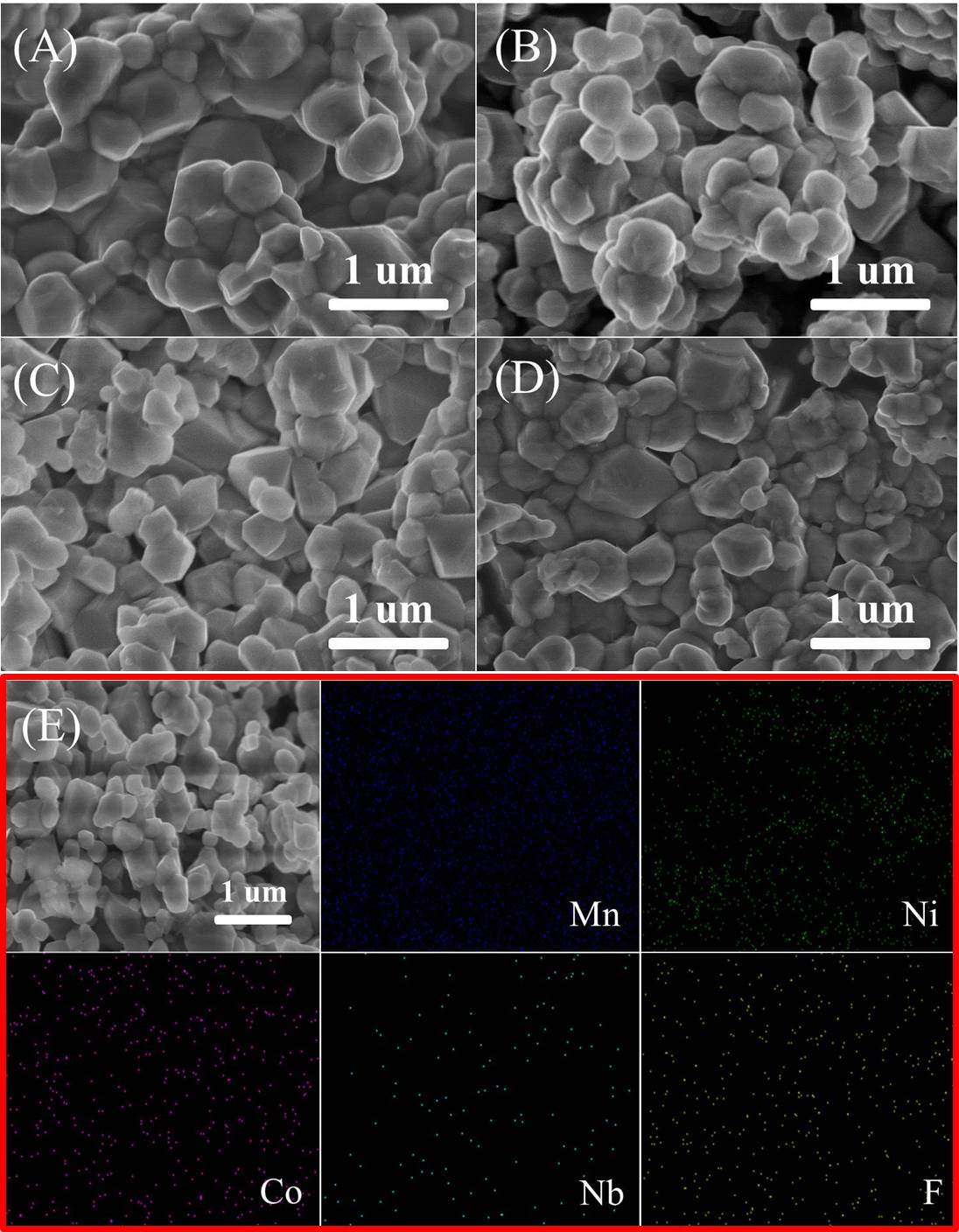


**Fig. S2.** SEM images of (A) LMNCO-NF0, (B) LMNCO-NF1, (C) LMNCO-NF3, and (D) LMNCO-NF5, and EDS elemental mapping (E) of LMNCO-NF3.

**Table S3.** The tap density of pristine LMNCO and all doped samples (LMNCO-NF1, LMNCO-NF3 and LMNCO-NF5)

| Samples | Tap density(g/cm^3^) |
| --- | --- |
| Pristine LMNCO | 1.51 |
| LMNCO-NF1 | 1.50 |
| LMNCO-NF3 | 1.52 |
| LMNCO-NF5 | 1.49 |

**Table S4.** The dissolution of Mn for pristine LMNCO and all doped samples (LMNCO-NF1, LMNCO-NF3 and LMNCO-NF5) after 100 and 200 cycles in the voltage range 2.0-4.8 V vs. Li/Li^+^ estimated by ICP analysis based on metal weight in the cathode.

| Samples | Amount of Manganese metal dissolved / wt% | |
| --- | --- | --- |
|  | After 100 cycles | After 200 cycles |
| LMNCO-NF0 | 3.02 | 3.95 |
| LMNCO-NF1 | 1.41 | 1.74 |
| LMNCO-NF3 | 1.34 | 1.55 |
| LMNCO-NF5 | 1.30 | 1.49 |

**Table S5**. Bond dissociation energies of metal oxygen bonds

| M | M-O bond energy ∆H_298_^a^ (KJ/mol) |
| --- | --- |
| Li | 341 |
| Ni | 391.6 |
| Co | 360 |
| Mn | 402 |
| Nb | 753 |
| ^a^ Bond dissociation energies are defined as the standard enthalpy change for the reaction MO=M+O at 298 K | |

**Table S6.** Comparison on the electrochemical performance of Li-rich layered oxides Li_1.2_Mn_0.54_Ni_0.13_Co_0.13_O_2_ in different reference.

| Samples | Initial coulombic efficiency | Rate performance: Capacity/rate  (mAh g^-1^/C) | Cycling performance: Capacity retention/rate/cycles  (%/C/n) | Ref. |
| --- | --- | --- | --- | --- |
| ZrO_2_ coated Li_1.2_Mn_0.54_Ni_0.13_Co_0.13_O_2_ | 77.8% | 253.1/0.1 | 94.9%/0.5/50 | 32 |
|  |  | 230.2/0.2 |  |  |
| Mg doped Li_1.2_Mn_0.54_Ni_0.13_Co_0.13_O_2_ | 72.8% | 275.8/0.2 | 92.4%/0.2/50 | 33 |
|  |  | 160.5/5 |  |  |
| Samaria doped ceria coated Li_1.2_Mn_0.54_Ni_0.13_Co_0.13_O_2_ | 77.9% | 261/0.1 | 89.3%/0.2/50 | 34 |
|  |  | 233/0.2 |  |  |
| LiNi_0.5_Mn_1.5_O_4_ coated Li_1.2_Mn_0.54_Ni_0.13_Co_0.13_O_2_ | 69.2% | 225/0.2 | 87.7%/1/60 | 35 |
|  |  | 95/5C |  |  |
| CeF_3_ coated  Li_1.2_Mn_0.54_Ni_0.13_Co_0.13_O_2_ | 80.8% | 222.9/0.1 | 91.7%/0.1/50 | 36 |
|  |  | 103.1/5 |  |  |
| LMNCO-NF3 | 81.4% | 269.8/0.1 | 98.1%/1/200 | This work |
|  |  | 173.3/5 |  |  |


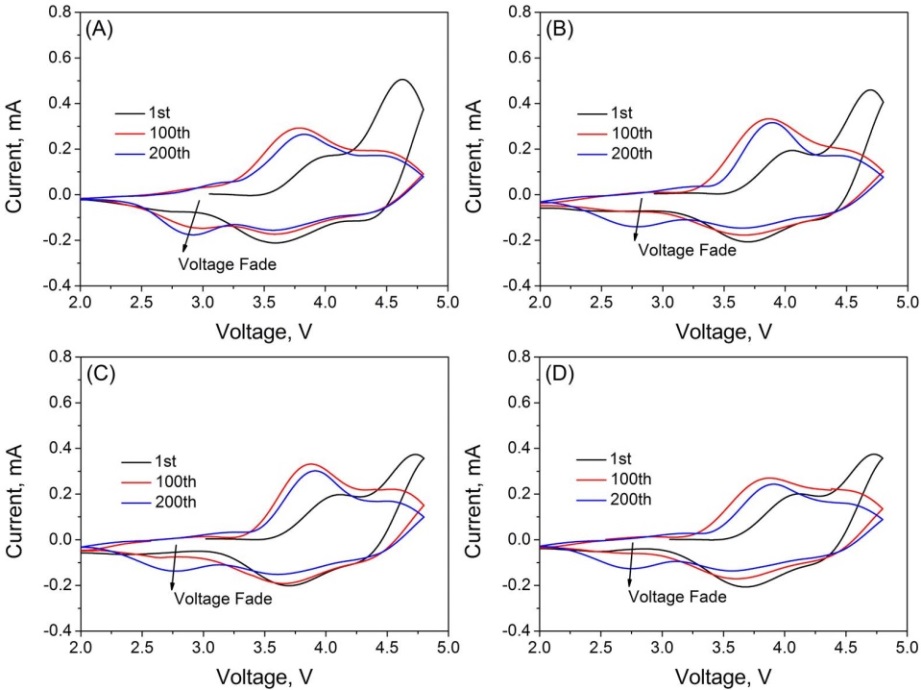


**Fig. S3.** Cyclic voltammograms of (A) LMNCO-NF0, (B) LMNCO-NF1, (C) LMNCO-NF3 and (D) LMNCO-NF5 materials before cycle for the initial cycle and after different cycles of 100th and 200th at 1 C.


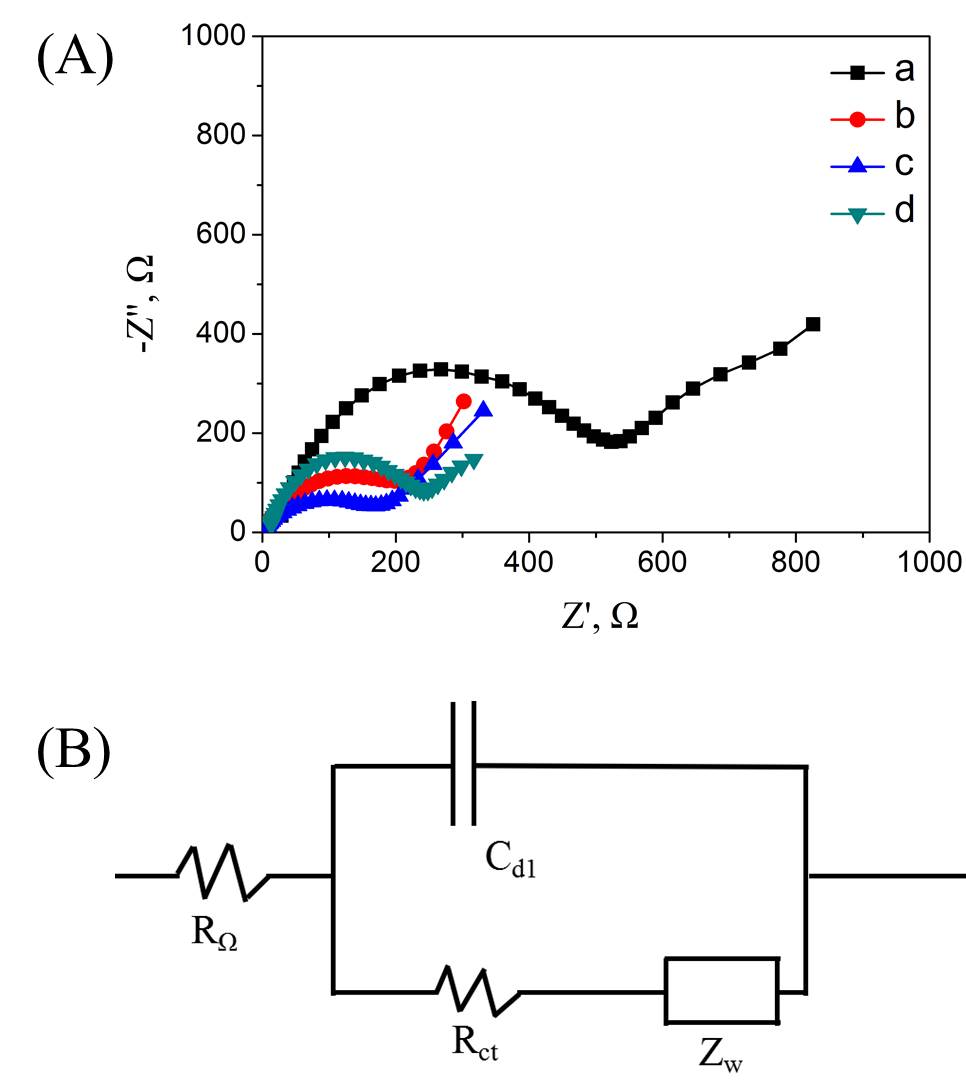


**Fig. S4.** (A) Nyquist plots of (a) LMNCO-NF0, (b) LMNCO-NF1, (c) LMNCO-NF3 and LMNCO-NF5, respectively. (B) Equivalent circuit used to analyze the impedance spectra of all samples before cycling and after different cycles at 1 C.

**Table S7**. Impedance parameters derived using equivalent circuit model (Fig. S5B) for LMNCO-NF0, LMNCO-NF1, LMNCO-NF3 and LMNCO-NF5 electrodes after cycling (fully discharged).

| Samples | LMNCO-NF0 | | | LMNCO-NF1 | | | LMNCO-NF3 | | | LMNCO-NF5 | | |
| --- | --- | --- | --- | --- | --- | --- | --- | --- | --- | --- | --- | --- |
|  | R_Ω_/Ω | R_ct_/Ω | D_Li_^+^/cm^2^s^-1^ | R_Ω_/Ω | R_ct_/Ω | D_Li_^+^/cm^2^s^-1^ | R_Ω_/Ω | R_ct_/Ω | D_Li_^+^/cm^2^s^-1^ | R_Ω_/Ω | R_ct_/Ω | D_Li_^+^/cm^2^s^-1^ |
| 1st | 8.6 | 596.3 | 0.83×10^−11^ | 5.0 | 227.1 | 2.18×10^−11^ | 4.8 | 205.4 | 2.41×10^−11^ | 5.5 | 285.3 | 1.74×10^−11^ |
| 100th | 10.3 | 888.3 | 0.58×10^−11^ | 5.8 | 283.2 | 1.75×10^−11^ | 5.2 | 240.3 | 2.06×10^−11^ | 6.3 | 338.6 | 1.46×10^−11^ |
| 200th | 13.4 | 1120.3 | 0.44×10^−11^ | 6.3 | 363.2 | 1.36×10^−11^ | 5.9 | 277.7 | 1.79×10^−11^ | 7.1 | 394.8 | 1.25×10^−11^ |
